# Bisphenol A Diglycidyl Ether (BADGE) and Progesterone Do Not Induce Ca^2+^ Signals in Boar Sperm Cells

**DOI:** 10.3389/fphys.2020.00785

**Published:** 2020-07-07

**Authors:** Anders Rehfeld, Noelia Mendoza, Raquel Ausejo, Niels Erik Skakkebæk

**Affiliations:** ^1^Department of Growth and Reproduction, Copenhagen University Hospital, Rigshospitalet, Copenhagen, Denmark; ^2^International Center for Research and Research Training in Endocrine Disruption of Male Reproduction and Child Health (EDMaRC), Rigshospitalet, University of Copenhagen, Copenhagen, Denmark; ^3^Department of Research and Development, Magapor SL, Zaragoza, Spain

**Keywords:** Endocrine disruption, fertility, CatSper, boar sperm, bisphenol

## Abstract

**Aim:**

Exposure of boar sperm cells to Bisphenol A diglycidyl ether (BADGE) has been shown to lead to reproductive failure in sows, however, the mode of action is unknown. As we have recently shown that BADGE can interfere with Ca^2 +^ signaling in human sperm cells through an action on CatSper, and as CatSper has been shown to be expressed in boar sperm cells, we hypothesized that a similar mechanism in the boar sperm cells could be responsible for the reproductive failure.

**Methods:**

Direct effects of BADGE and the endogenous ligand of human CatSper, progesterone, on Ca^2+^ signaling in human and boar sperm cells were evaluated side-by-side using a Ca^2+^ fluorimetric assay measuring changes in intracellular Ca^2+^. Effects of BADGE on Ca^2+^ signaling in boar sperm were furthermore assessed by flow cytometry by an independent laboratory.

**Results:**

The exact same solutions of BADGE and progesterone induced transient biphasic Ca^2+^ signals in human sperm cells, but failed to do so in both non-capacitated and capacitated boar sperm cells. BADGE also failed to induce transient biphasic Ca^2+^ signals in boar sperm cells in the flow cytometric assay.

**Conclusion:**

BADGE and progesterone failed to induce Ca^2+^ signals in boar sperm cells. This indicates that the signaling mechanisms leading to activation of CatSper differs between human and boar sperm cells, and suggests that the mode of action by which exposure of boar sperm cells to BADGE can lead to reproductive failure in sows does not involve effects on Ca^2+^ signaling.

## Introduction

The CatSper Ca^2+^ channel is a sperm specific Ca^2+^ channel highly conserved in mammals (Cai and Clapham, 2008), but also present in a wide range of other species (Romero and Nishigaki, 2019). Ca^2+^ signaling is a key regulator of sperm function and CatSper thus controls important sperm functions (Lishko et al., 2012). In human (Lishko et al., 2011; Strünker et al., 2011) and macaque sperm cells (Sumigama et al., 2015) CatSper has been shown to be activated by the female sex steroid progesterone, released in high amounts from the cumulus cells surrounding the oocyte (Lishko et al., 2011; Strünker et al., 2011). However, in mouse sperm cells progesterone fails to activate CatSper (Lishko et al., 2011; Schiffer et al., 2014), hinting that signaling pathways leading to CatSper activation may be more different than similar, even between mammalian species (Kaupp and Strünker, 2017). CatSper has been shown to be expressed in boar sperm cells (Song et al., 2011; Vicente-Carrillo et al., 2017), and has been suggested to be functional through the use of CatSper-inhibitors (Vicente-Carrillo et al., 2017; Machado et al., 2019), but the exact role of CatSper in boar sperm cells remains unclear.

Bisphenol A diglycidyl ether (BADGE) is synthesized through O-alkylation of bisphenol A (BPA) with epichlorohydrin and is a widely used constituent of, e.g., epoxy resins, paints, and food container linings (Chamorro-García et al., 2012). Recently, a study found that BADGE could leach from plastic bags used for storage of boar semen and that exposure of boar sperm cells to BADGE led to reproductive failure in sows (Nerin et al., 2014), however, without any clear mode of action identified. As we have shown that BADGE in μM concentrations can induce transient biphasic Ca^2+^ signals via an activation of CatSper in human sperm cells (Rehfeld et al., 2020), we hypothesized that a similar mechanism in the boar sperm cells could be responsible for the reproductive failure in sows. Here we set out to test this hypothesis, by investigating whether BADGE could interfere with Ca^2+^ signaling in boar sperm cells through an examination of the effect of both BADGE and the endogenous ligand of human CatSper, progesterone, on human and boar sperm cells side-by-side using a Ca^2+^ fluorimetric assay.

## Materials and Methods

### Chemicals and Reagents

Bisphenol A diglycidyl ether (BADGE) was purchased from Sigma-Aldrich (St. Louis, MO, United States) and dissolved in DMSO at a stock concentration of 10 mM. Progesterone, A23187 and ionomycin were obtained from Sigma-Aldrich (St. Louis, MO, United States) and dissolved in DMSO at stock concentrations of 20 mM, 100 mM and 1 mM, respectively. Fluo-4, AM, was purchased from Invitrogen (Carlsbad, CA, United States). Fluo-3, AM, and propidium iodide were obtained from Sigma-Aldrich (St. Louis, MO, United States). Human serum albumin was obtained from Irvine Scientific (Santa Ana, CA, United States). Dulbecco’s Phosphate Buffered Saline with calcium chloride and magnesium chloride (DPBS+) (Item # D8662) and Dulbecco’s Phosphate Buffered Saline without calcium chloride and magnesium chloride (DPBS-) (Item # D8537) were obtained from Sigma-Aldrich (St. Louis, MO, United States).

### Semen Samples

Human semen samples from volunteer donors were produced by masturbation and ejaculated into wide-mouthed plastic containers, on the same day as the experiment and allowed to liquefy for 15–30 min at 37°C. The volunteer donors were recruited from the semen donor corps, which is routinely donating samples for quality control analyses at the Department of Growth and Reproduction, Rigshospitalet. All volunteers fulfilled WHO criteria for normal semen quality. Each experimental replicate was based on sperm cells from a single sperm sample.

Boar semen samples for the Ca^2+^ fluorimetric assay were produced the day before the experiment and obtained as raw semen samples from Ringsted Forsøgslaboratorium, Denmark, a part of Hatting A/S. For the flow cytometry experiments eight ejaculates were collected on the same day as the experiment from eight different animals in different Spanish boar studs, diluted 1:10 in commercial boar semen extender Duragen^®^ and then immediately sent to Magapor SL laboratories. The viability was evaluated by flow cytometry using propidium iodide staining and motility was evaluated using a commercial computer assisted sperm analysis system (CASA) (ISAS Proiser, Spain) as in (Nerin et al., 2014).

### Purification of Motile Sperm Cells via Swim-Up

For the Ca^2+^ fluorimetric assay, motile sperm cells were isolated from human and boar semen samples by the swim-up method (Rehfeld et al., 2019). Briefly 1 mL of semen was gently placed in the bottom of 50 mL tube containing 4 mL of human tubular fluid (HTF^+^) medium with the composition: 97.8 mM NaCl, 4.69 mM KCl, 0.2 mM MgSO_4_, 0.37 mM KH_2_PO_4_, 2.04 mM CaCl_2_, 0.33 mM Na-pyruvate, 21.4 mM Na-lactate, 2.78 mM glucose, 21 mM HEPES, and 4 mM NaHCO_3_, adjusted to pH 7.3–7.4 with NaOH. After 1 h at 37°C, the upper swim-up fraction was carefully removed and after two washes, the sperm concentration was determined by image cytometry (Egeberg et al., 2013) using an NC-3000 (ChemoMetec AS, Denmark) and samples were adjusted to 10 × 10^6^ sperm cells/mL in HTF^+^ with human serum albumin (3 mg/mL). Hereafter the sperm cells were incubated for at least 1 h at 37°C. For the experiments with capacitated boar sperm cells, the samples were treated similar to in a previous study on boar sperm (Bernecic et al., 2019) and resuspended in a capacitating medium with the following composition: 72.8 mM NaCl, 4.69 mM KCl, 0.2 mM MgSO_4_, 0.37 mM KH_2_PO_4_, 2.04 mM CaCl_2_, 0.33 mM Na-pyruvate, 21.4 mM Na-lactate, 2.78 mM glucose, 21 mM HEPES, and 25 mM NaHCO_3_, adjusted to pH 7.3–7.4 with NaOH. Human serum albumin (3 mg/mL) was added to the capacitating medium and the sperm cells were incubated for >3 h at 37°C in a 5% CO_2_ atmosphere.

For the flow cytometry experiments the boar semen samples diluted 1:10 in extender, were simply diluted further to a final concentration of 4 × 10^7^ cells per mL in DPBS+.

### Measurement of Changes in [Ca^2+^]_i_ in Ca^2+^ Fluorimetric Assay

Changes in the free intracellular Ca^2+^ concentration [Ca^2+^]_i_ in human and boar sperm cells were measured in 384 multi-well plates in a fluorescence plate reader (Fluostar Omega, BMG Labtech, Germany) at 30°C as described in Rehfeld et al. (2019). Briefly, sperm cells were incubated with the fluorescent Ca^2+^ indicator Fluo-4, AM (10 μM) for 45 min at 37°C. Excess dye was removed by centrifugation (700 × *g*, 10 min, RT) and the sperm pellet was resuspended in HTF^+^ to 5 × 10^6^ sperm cells/mL. Just before loading the sperm cells to the 384-well plates, sperm motility was evaluated manually using phase contrast optics on an Olympus BX45 microscope at a total magnification of ×200 (Olympus, Denmark) to make sure that the sperm cells used for the experiments were motile and thus viable. Aliquots of 50 μL were loaded to the wells of a 384-well plate using an automatic repeater pipette. Fluorescence was excited at 480 nm and emission was recorded at 520 nm with bottom optics. Fluorescence was recorded before and after addition of 25 μL bisphenol solutions, negative control (buffer with vehicle), and positive controls (progesterone, 5 μM final concentration, and ionomycin, 10 μM final concentration) manually with an electronic multichannel pipette to duplicate wells. Changes in Fluo-4 fluorescence are shown as ΔF/F_0_ (%), indicating the percentage change in fluorescence (ΔF) with respect to the mean basal fluorescence (F_0_) before addition of BADGE, positive control and negative control.

### Measurement of Changes in [Ca^2+^]_i_ Using Flow Cytrometry

Changes in [Ca^2+^]_i_ in boar sperm cells were additionally measured using Fluo-3 and flow cytometry, similar to what other have previously used in boar sperm cells (Schmid et al., 2013; Yeste et al., 2015). The measurements were performed on a BD Accuri^TM^ C6 (Becton Dickinson, Madrid, Spain) with BD software. At least 40,000 events were counted in every measurement. Sperm population was gated for further analysis on the basis of its specific forward (FS) and side scatter (SS) properties; other non-sperm events were excluded. To stain the boar sperm cells 3.5 μL of Fluo-3, AM stock solution (2 mM in DMSO) was added to 400 μL of sperm samples (4 × 10^7^ cells per mL), giving a final Fluo-3, AM, concentration of 17.5 μM and incubated for 45 min at 37°C protected from light. After the incubation, small aliquots of the BADGE stock solution were added to each sample, respectively, yielding final BADGE concentrations of 100, 50, 25, 12.5, 6.25, 3.125, and 1.562 μM. As a positive control the calcium ionophore A23187 was added at 1 mM final concentration to one of the samples and as a negative control DPBS- buffer was added. Just after adding BADGE, the positive control or the negative control, samples were measured in the flow cytometer at different times. Changes in Fluo-3 fluorescence are shown as ΔF/F_0_ (%), indicating the percentage change in fluorescence (ΔF) with respect to the mean basal fluorescence (F_0_) before addition of BADGE, positive control and negative control.

### Statistical Analyses

Comparison of peak Ca^2+^ signal amplitudes were done using one-way ANOVA. *P*-values were corrected for multiple comparison type I error inflation by Dunnett’s method. Statistical analyses were performed using GraphPad Prism 8.3.1 (GraphPad Software Inc., United States).

### Ethical Approval

Healthy human volunteers donated the semen samples after their prior consent. The volunteers were recruited from the semen donor corps, which is routinely donating samples for quality control analyses at the Department of Growth and Reproduction, Rigshospitalet. After delivery, the samples were fully anonymized and no data on the fertility status or general health of donors is provided. Each donor received a fee of 500 DKK (about 75 UD dollars) per sample for their inconvenience. All samples were analyzed on the same day of delivery and destroyed immediately after the laboratory analyses. Because of the full anonymization of the samples, and the destruction of the samples immediately after the laboratory analyses, no ethical approval was needed for this work, according to the regional scientific ethical committee of the Capital Region of Denmark.

## Results

### Effects of BADGE and Progesterone on Ca^2+^ Signaling in Boar Sperm Cells

**FIGURE 1:**
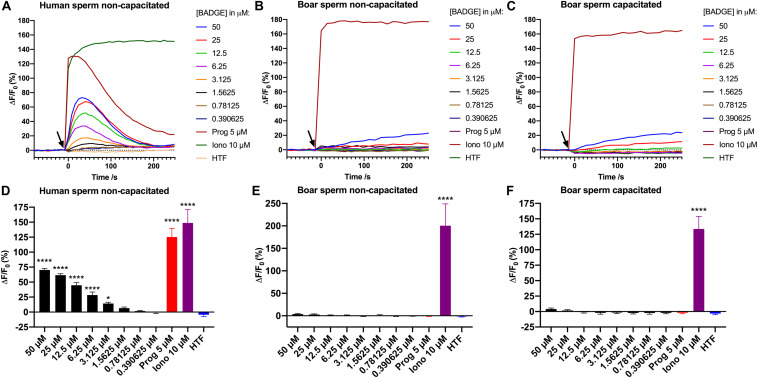
Ca^2+^ signals induced by addition of serially diluted doses of BADGE, 5 μM progesterone, 10 μM ionomycin, and a negative buffer control “HTF” to **(A)** non-capacitated human sperm cells, **(B)** non-capacitated boar sperm cells, and **(C)** capacitated boar sperm cells. The black arrow depicts the time of addition of solutions to the sperm cells. Graphs **(A–C)** are from single representative experiments. Mean amplitude of the induced Ca^2+^ signals 30 s after addition of compounds and controls are shown for **(D)** non-capacitated human sperm cells (*n* = 4), **(E)** non-capacitated boar sperm cells (*n* = 6), and **(F)** capacitated boar sperm cells (*n* = 3). Statistics are from one-way ANOVA analyses comparing the mean amplitude of the induced Ca^2+^ signals 30 s with the mean amplitude of the Ca^2+^ signal induced by HTF buffer alone. **** depicts an adjusted *p*-value of <0.0001, and * depicts an adjusted *p*-value of 0.0286.

We investigated BADGE for its ability to induce Ca^2+^ signals in human and boar sperm cells, using a Ca^2+^ fluorimetric assay (Schiffer et al., 2014). BADGE was tested at decreasing serially diluted concentrations from a starting concentration of 50 μM, along with the endogenous ligand of human CatSper, progesterone, at 5 μM, ionomycin at 10 μM, and a negative buffer control (HTF^+^). Changes in [Ca^2+^]_i_ were recorded for 250 s after addition of the compounds. Our results showed that addition of the exact same solutions of BADGE and progesterone to the sperm cells, induced transient biphasic Ca^2+^ signals in the human sperm cells, but failed to do so both in non-capacitated and capacitated boar sperm cells (*n* ≥ 3) (Figures 1A–C), whereas addition of 10 μM ionomycin induced rapid and saturating Ca^2+^ signals in both human and boar sperm cells. In boar sperm, only a small, slowly rising Ca^2+^ signal was induced by 50 and 25 μM of BADGE, but these Ca^2+^ signals did not resemble the transient biphasic Ca^2+^ signal induced by BADGE in human sperm cells. When comparing the amplitude of the induced Ca^2+^ signals 30 s after addition of compounds, a time point where both the progesterone- and BADGE-induced Ca^2+^ signals peak in human sperm cells, we found that BADGE at concentrations ≥3,125 μM induced Ca^2+^ signals significantly larger than those induced by HTF buffer alone in human sperm cells (Figure 1D). 5 μM progesterone and 10 μM ionomycin, similarly induced significantly larger Ca^2+^ signals in human sperm cells (Figure 1D). In contrast to this, only 10 μM ionomycin induced Ca^2+^ signals significantly larger than those induced by HTF buffer alone in both non-capacitated and capacitated boar sperm cells (Figures 1E,F).

### Effects of BADGE on Ca^2+^ Signaling in Boar Sperm Cells in an Independent Laboratory

**FIGURE 2:**
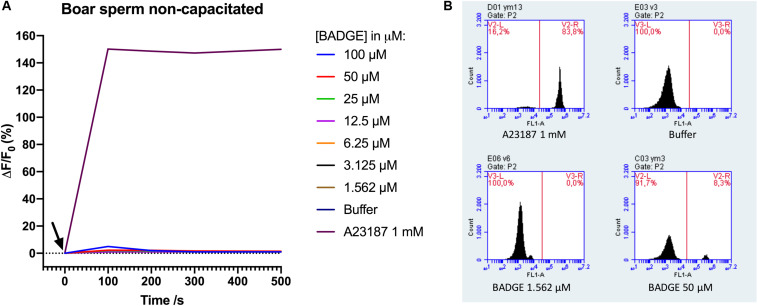
**(A)** Ca^2+^ signals measured by flow cytometry after addition of serially diluted doses of BADGE, 1 mM of ionophore A23187, and a negative buffer control (DPBS-) to non-capacitated boar sperm cells. The black arrow depicts the time of addition of solutions to the sperm cells. Representative data from a single experiment. **(B)** representative flow cytometric Fluo-3 fluorescence images from a single experiment.

To scrutinize our negative results for BADGE above, we contacted an independent laboratory to get them to repeat the experiment. They similarly tested BADGE for effects on Ca^2+^ signaling in boar sperm cells using a slightly different experimental setup. Using boar sperm of a diluted, raw boar semen sample instead of swim-up purified sperm, a flow cytometer instead of a plate reader, and the Ca^2+^-fluorophore Fluo-3 instead of Fluo-4, BADGE was again tested at decreasing serially diluted concentrations from a starting concentration of 100 μM, along with a positive control, ionophore A23187 at 1 mM, and a negative buffer (DPBS-) control. The data from these experiments were almost identical to our initial results from the Ca^2+^ fluorimetric plate reader based assay. BADGE at any concentration failed to induce Ca^2+^ signal in non-capacitated boar sperm cells (*n* = 8) (Figure 2), whereas addition of 1 mM A23187 induced rapid and saturating Ca^2+^ signal in the boar sperm cells.

## Discussion

A recent study showed that exposure of boar sperm cells to BADGE could lead to reproductive failure in sows (Nerin et al., 2014), although the mode of action remained unknown. Intriguingly, no effects were found on routine boar sperm parameters, including motility and viability, or on in vitro fertilization tests, but only on in vivo fertility rates in sows (Nerin et al., 2014). We speculated that the reproductive failure could be due to effects on Ca^2+^ signaling through an action on CatSper in the boar sperm cells, as CatSper has been shown to be expressed in boar sperm cells (Song et al., 2011; Vicente-Carrillo et al., 2017), has been suggested to be functional through the use of CatSper-inhibitors (Vicente-Carrillo et al., 2017; Machado et al., 2019), and as we have recently found that BADGE in μM concentrations can interfere with Ca^2+^ signaling through an action on CatSper in human sperm cells (Rehfeld et al., 2020). Our results here, however, showed that addition of the exact same solutions of BADGE to the sperm cells, induced transient biphasic Ca^2+^ signals in the human sperm cells, but failed to do so both in non-capacitated and capacitated boar sperm cells (*n* ≥ 3) (Figure 1), similar to after addition of 5 μM progesterone. Furthermore, a similar experiment in an independent laboratory, using a slightly different experimental setup, confirmed the lacking ability of BADGE to induce transient biphasic Ca^2+^ signals in boar sperm cells (Figure 2). As the sperm cells were motile and thus viable just prior to running the Ca^2+^ fluorimetric assay experiments, the difference in the Ca^2+^ responses between human and boar sperm cells are unlikely to be caused by a lack of viable boar sperm cells. In line with this, the very large Ca^2+^ signal induced by the Ca^2+^-ionophores ionomycin (Figure 1) and A23187 (Figure 2) indicates that, at the moment of adding the ionophones to the sperm cells, a large proportion of the sperm cells must have been viable, as unviable sperm cells cannot maintain their Ca^2+^ gradient across the cell membrane. Our findings therefore do not support our hypothesis that exposure of boar sperm cells to BADGE leads to reproductive failure in sows (Nerin et al., 2014) through an effect on Ca^2+^ signaling in boar sperm cells, similar to the effect that we have recently shown for BADGE on human sperm cells (Rehfeld et al., 2020).

Interestingly, the structurally similar compound BPA, has been shown to inhibit mouse CatSper transiently in low pM-nM concentrations and to cause a significant reduction in motility and acrosome reaction in mouse sperm cells (Wang et al., 2016). A similar inhibitory action of BADGE on boar CatSper could take place, although we did not observe a large decrease in Fluo-4 or Fluo-3 fluorescence [ΔF/F_0_ (%)] (Figures 1, 2) after application of BADGE, similar to what has been shown after addition of the potent CatSper inhibitor RU1968 to human sperm cells (Rennhack et al., 2018). Future studies will have to examine this using electrophysiological measurements on boar sperm cells.

Furthermore, our finding that the same solution of progesterone (5 μM) induced a large biphasic Ca^2+^ signals in the human sperm cells, but failed to do so both in non-capacitated and capacitated boar sperm cells (*n* ≥ 3) (Figure 1) also do not support the hypothesis that boar CatSper should be activated by progesterone as seen for human CatSper, as has been suggested by others (Machado et al., 2019). The fact that 17-OH-progesterone (Strünker et al., 2011) and pregnenolone sulfate (Mannowetz et al., 2017; Brenker et al., 2018) are potent ligands of human CatSper, but that both 17-OH-progesterone and pregnenolone did not mimic the action of progesterone in boar sperm cells (Machado et al., 2019), further fails to support a similar mechanism of activation between human and boar CatSper. It is possible that boar sperm cells need to undergo some form of maturation process, other than capacitation, before boar CatSper can be activated by progesterone. Future studies will have to examine this. However, as human CatSper can be activated by progesterone even in testicular and epididymal human sperm cells (Smith et al., 2013), without the sperm cells having to go through any form of maturation process first, this would again suggest large differences in the events leading to activation of CatSper activation between human and boar sperm cells. Even though CatSper has been shown to be expressed in boar sperm cells (Song et al., 2011; Vicente-Carrillo et al., 2017), and has been suggested to be functional through the use of CatSper-inhibitors (Vicente-Carrillo et al., 2017; Machado et al., 2019), electrophysiological evidence of CatSper-conductance in boar sperm cells need to be obtained to confirm a possible functional role in this species. To our knowledge, such data are yet to be published.

We are unaware of any studies showing induction of transient biphasic Ca^2+^ signal by progesterone in physiological (low μM) concentrations in boar sperm cells, although, studies have found an induction of transient Ca^2+^ signals by progesterone at very high concentrations (100 μM) (Kim et al., 2008) and (10 μg/mL or 31.8 μM) (Yeste et al., 2015), similar to what has been seen in mouse sperm cells after addition of progesterone at very high concentrations (40 and 100 μM) (Romarowski et al., 2016). As mouse CatSper is not activated by progesterone (Lishko et al., 2011; Schiffer et al., 2014), these Ca^2+^ signals must be induced by some other mode of action, which is supported by the finding that progesterone at 1 mM can even induce Ca^2+^ signals in CatSper^–/–^ mouse sperm cells (Ren et al., 2001). However, progesterone at a much lower nM concentrations has been shown to affect boar sperm penetration through a cell separation media (100 nM) (Campbell, 2013), to affect the acrosome reaction in capacitated boar sperm cells (100 nM) (Campbell, 2013), to affect the release of boar sperm cells from oviductal cells (80 nM) (Machado et al., 2019), and to induce a slow increase in intracellular Ca^2+^ evident after 30 min of incubation (80 nM) (Machado et al., 2019), probably associated with an induction of capacitation. Furthermore, a progesterone gradient from a starting concentration of 1 μM has been shown to act chemotactically on boar sperm cells (Berendsen et al., 2020). It is likely that progesterone exerts these effects in boar sperm cells through a mechanism unrelated to Ca^2+^ signaling and CatSper. If this is the case, BADGE could possibly have caused the reproductive failure through this same unknown pathway. Future studies are needed to clarify this. Importantly, our findings are in line with other studies showing that the activation of CatSper by progesterone is unique to human (Lishko et al., 2011; Strünker et al., 2011) and primate sperm cells (Sumigama et al., 2015), whereas mouse CatSper is insensitive to both the chemicals affecting human CatSper (Schiffer et al., 2014) and to progesterone (Lishko et al., 2011). This means that observations on reproductive toxicology in non-primate animal models cannot simply be translated to humans in terms of effects on CatSper-mediated sperm function and consequently in terms of effects on fertility.

## Conclusion

In conclusion, our study fails to support our hypothesis that exposure of boar sperm cells to BADGE leads to reproductive failure in sows (Nerin et al., 2014) through an effect on Ca^2+^ signaling in boar sperm cells. Furthermore, our data do not support the hypothesis by others (Machado et al., 2019) that boar CatSper can be activated by progesterone as seen for human CatSper. Future studies will have to validate our results and further explore the mode of action by which exposure of boar sperm cells to BADGE can lead to reproductive failure in sows (Nerin et al., 2014), as this could be of high interest given the widespread human exposure to BADGE (Chamorro-García et al., 2012).

## Data Availability Statement

The raw data supporting the conclusions of this article will be made available by the authors, without undue reservation.

## Ethics Statement

Ethical review and approval was not required for the study on human participants in accordance with the local legislation and institutional requirements. The patients/participants provided their written informed consent to participate in this study. Ethical review and approval was not required for the animal study because we only used anonymous boar sperm samples from a commercial provider.

## Author Contributions

AR and NS conceived the study and drafted the manuscript. AR designed, planned, and performed the experiments on the plate reader. NM and RA designed, planned, and performed the experiments on the flow cytometer. All authors contributed to the article and approved the submitted version.

## Conflict of Interest

RA and NM were employed by the company Magapor SL. The remaining authors declare that the research was conducted in the absence of any commercial or financial relationships that could be construed as a potential conflict of interest.
